# Outdoor mm-wave 5G/6G transmission with adaptive analog beamforming and IFoF fronthaul

**DOI:** 10.1038/s41598-023-40112-w

**Published:** 2023-08-25

**Authors:** Javier Pérez Santacruz, Elmine Meyer, Roel X. F. Budé, Catalina Stan, Antonio Jurado-Navas, Ulf Johannsen, Idelfonso Tafur Monroy, Simon Rommel

**Affiliations:** 1 Department of Electrical Engineering, Eindhoven University of Technology, 5600MB Eindhoven, The Netherlands; 2 Telecommunication Research Institute (TELMA), Universidad de Málaga, 29010 Málaga, Spain

**Keywords:** Electrical and electronic engineering, Fibre optics and optical communications, Microwave photonics

## Abstract

Adaptive analog beamforming is a key technology to enable spatial control of millimeter-wave wireless signals radiated from phased array antennas (PAAs) which is essential to maximize the capacity of future mobile networks and to ensure efficient usage of scarce spectrum. Intermediate frequency-over-fiber (IFoF), on the other hand, is a promising technology for the millimeter-wave (mm-wave) mobile fronthaul due to its low complexity, high optical spectral efficiency, and low latency. The combination of IFoF and PAA is key to implement mm-wave mobile communications in a scalable, centralized, efficient, and reliable manner. This work presents, for the first time to the best of the authors’ knowledge, an extensive outdoor measurement campaign where an experimental IFoF mm-wave wireless setup is evaluated by using PAAs with adaptive beamforming on the transmitter and receiver sides. The configuration of the experimental setup is according to 5G standards, transmitting signals wirelessly at 27 GHz central frequency in the n258 band. The employed PAAs are composed of 8-by-8 patch antenna arrays, allowing beam steering in the azimuth and elevation angles. Furthermore, different end-user locations, antenna configurations, and wireless scenarios are tested in the outdoor experiment, showing excellent EVM performance and achieving 64-QAM transmission over up to 165.5 m at up to 1.88 Gbit/s. The experimental results enable optimization of the experimental setup for different scenarios and prove the system’s reliability in different wireless conditions. In addition, the results of this work prove the viability and potential of IFoF combined with PAA to be part of the future 5G/6G structure.

## Introduction

The dramatic growth of mobile data traffic in the last years requires a major upgrade and enhancement of the network infrastructure, especially with the emergence of new applications and services such as augmented reality (AR), virtual reality (VR), 4K/8K video streaming, autonomous driving, Industry 4.0, and Internet of Things (IoT)^1^. The fifth-generation (5G) of mobile networks and its successor 6G aim to provide an adequate quality of experience (QoE) and quality of service (QoS) for such applications. To achieve this, 5G standards specify a set of requirements in terms of latency, number of connected devices, data rate, energy efficiency, mobility, and capacity^2^. Increasing the data rate is one of the main objectives of future 5G/6G systems. To realize this, it is necessary to move towards higher frequency bands. Optical wireless communications provide a vast amount of available bandwidth to exploit. However, optical wireless communications offer low reliability, small coverage areas, and low sensibility, making them unsuitable for outdoor mobile scenarios^3^. In contrast, millimeter-wave (mm-wave) wireless communications overcome the drawbacks of the optical approach, permitting the use of significant amounts of available spectrum. One of the most important challenges in mm-wave wireless communications is the power limitation due to the increased free-space path loss (FSPL), atmospheric attenuation, and penetration losses^4^.

Beamforming is a key method to alleviate the power limitations due to high path loss in mm-wave wireless communications^5^ and to enable spatial control over the signal, minimizing interference and allowing increased frequency re-use. While digital beamforming is most commonly applied in low-bandwidth sub-7 GHz systems, it requires a full RF chain, analog-to-digital converters (ADCs) and digital-to-analog converters (DACs) for each antenna element, making it scale poorly in terms of cost, complexity and power consumption, especially for mm-wave massive multiple-input and multiple-output (MIMO) applications^5, 6^. As a consequence, analog and hybrid beamforming technologies enable the deployment of mm-wave mobile communications in an effective and scalable manner, since they reduce the number of required ADCs and DACs. In particular, phased array antennas (PAAs) are one of the most promising analog beamforming implementations, giving rapid and flexible beam steering capabilities^7, 8^.

From the architecture perspective, the implementation of mm-wave cells requires a reduction in the coverage radius ($\approx$ 200 m), in comparison with the current sub-7 GHz network. In other words, compared to current mobile networks, the expected number of mm-wave cells for future 5G/6G scenarios is vast^9, 10^. Compared to classical distributed radio access network (RAN) deployments, centralized radio access network (C-RAN) is the preferred architecture especially for mm-wave mobile deployments, due to its intrinsic benefits such as low maintenance, centralized monitoring, and reduced energy consumption^11^. C-RAN adds a new segment in the mobile infrastructure, called fronthaul which interconnects the central office (CO) with the remote antenna unit (RAU). Hence, radio-over-fiber (RoF) arises as an ideal technology to transport and distribute the data in the mobile fronthaul and reduce the complexity of the remote stations which is crucial for deployment of the large numbers of cells required^12–14^. There are three main types of RoF technologies technologies^15–17^: mm-wave analog radioover-fiber (ARoF), intermediate frequency-over-fiber (IFoF), and digital radio-over-fiber (DRoF). In fact, the two first RoF technologies are considered ARoF since they transport analog signal through the optical fiber. Current fronthaul solutions such as common public radio interface (CPRI) and next generation fronthaul interface (NGFI) employ DRoF. However, the mobile fronthaul is expected to become a bottleneck as it struggles to transport the enormous RF bandwidths required to support the growth of mobile data traffic and due to the low spectral efficiency of DRoF fronthaul technologies. On the other hand, ARoF is a suitable solution to solve the fronthaul bottleneck due to its high optical spectral efficiency and potentially the inclusion of beamforming in the optical domain, improving bandwidth and reducing beamformer footprint^18, 19^. Moreover, in contrast to the DRoF approach, ARoF solutions allow a great complexity reduction of the RAU, moving most of the functionalities to the CO. This low-complexity RAU feature is essential to scalably and efficiently deploy a large number of mm-wave cells for 5G/6G^13^, while reducing maintenance cost and latency^11^.

However, compared to DRoF, mm-wave ARoF may suffer from stronger signal degradations due to the non-ideal functionality of the components involved in RoF systems and the combined fiber (chromatic dispersion, nonlinear distortions) and wireless channel^16, 20^. IFoF avoids the use of optical heterodyning, i.e., the beating of two optical carriers for mm-wave generation, and potential resulting issues with phase noise and/or signal-signal beating, as experienced in mm-wave ARoF systems, while taking advantage of the benefits of analog fiber transport. Since IFoF dispenses with optical mm-wave transport or optical heterodyne, the optical complexity and the bandwidth requirements on optical components are reduced^15^, at the cost of requiring mm-wave upconversion and a local oscillator at the RAU site, which increases complexity, cost, and energy consumption. Table 1 compiles the three described fronthaul technologies in terms of signal degradation in the optical fiber and complexity of CO and RAU. Observing Table 1, it can be noted that IFoF brings intermediate features between the mm-wave ARoF and DRoF solutions, being a suitable choice to implement in many mobile scenarios^17^ and, combined with PAA-based beamforming, is a promising solution for future mm-wave 5G/6G mobile networks. Finally, sigma-delta-over-fiber (SDoF) which offers lower system complexity than DRoF and stronger signal integrity than ARoF technologies should be mentioned as a candidate RoF fronthaul technology. In contrast to ARoF, SDoF is characterized by a quantification noise which reduces the spectral efficiency in the optical fiber^21^.

**Table 1:** Comparison table of the three RoF fronthaul technologies.

Fronthaul technology	CO complexity	RAU complexity	Signal degradation level
mm-wave ARoF	High	Low	High
IFoF	Medium	Medium	Medium
DRoF	Medium	High	Low

Beamforming with PAAs and IFoF combine ideally to address some of the challenges associated with mm-wave mobile communications^5, 13, 22^, such as congestion in the fronthaul due to the increased data rates and larger bandwidths, and low received powers, especially in scenarios with line-of-sight (LOS) blockage. In previous works, the IFoF technique has been extensively studied as a 5G fronthaul solution, validating its efficiency for 28 GHz mm-wave communications^17^, and V-band systems implementing various modulation formats (quadrature phase shift keying (QPSK), quadrature amplitude modulation (QAM)) and PAA-based beam steering techniques^23–25^. The coexistence of IFoF signal with passive optical network (PON) traffic was successfully demonstrated in the field environment and evaluated by using 16-QAM orthogonal frequency-division multiplexing (OFDM) signals^26^, while from a fronthaul capacity perspective, IFoF has been used to experimentally demonstrate an aggregate capacity up to 24 Gbit/s over 7 km fiber and 5 m V-band link^27^. An outdoor experiment using IFoF with 28 GHz mm-wave wireless transmission and 16-QAM and 64-QAM modulation orders has been demonstrated^28^, with the terminal located at 10 m and 1 km LOS away from the remote radio head (RRH) in charge of beamforming using multiple fixed beams. Nevertheless, to the best of the authors’ knowledge, there are no scientific reports in the research literature regarding experimental mm-wave wireless IFoF 5G setups with PAA-based beam steering evaluated in outdoor scenarios. In fact, the majority of the previous experimental mm-wave optical fronthaul works are focused on indoor scenarios with fixed beam antennas. Thus, the presented work stands out for the combination of IFoF together with beam steerable antennas in an outdoor scenario. In particular, this manuscript presents an experimental wireless IFoF testbed realizing different outdoor scenarios at a center frequency of 27 GHz, within the n257 and n258 5G bands^2^. The presented outdoor experiment is performed in a parking lot, serving as a measurement campaign for evaluation and validation of vehicle applications such as remote driving or cooperative, connected and automated mobility (CCAM) services^29^. In the experimental setup, a pair of PAA panels are employed on the transmitter and receiver sides, allowing for beam steering capabilities. Furthermore, the experiment configuration is according to 5G standards, successfully transmitting 64-QAM OFDM signals with a subcarrier spacing of 240 kHz^2^.

The remainder of this manuscript is structured as follows: the second section describes the overall concept of the used architecture and the different wireless scenarios where the transmission performance is analyzed; the third section discusses the utilized PAA panels together with their characterization, details the experimental setup, and explains the digital signal processing (DSP) processes carried out to obtain the final results; in the fourth section, the experimental results are presented, analyzed, and interpreted; finally, the fifth section gives conclusive remarks concerning the contribution of the presented work.

## Wireless scenarios

**Figure 1:**
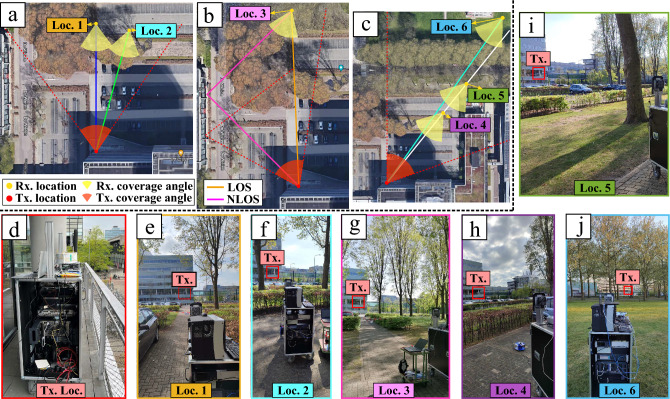
Locations and configurations of the different wireless scenarios utilized in the outdoor measurement campaign: (**a**–**c**) 2D maps representing the different measurement locations with their respective antenna orientations; (**d**) photo of the transmitter cart located on the first floor of Flux building; (**e**–**j**) photos of the different locations of the end-user cart where the transmitter cart can be seen.

Given the importance of high-speed communication in the future of the automotive industry, it is chosen to perform the outdoor experiment in the parking lot of building Flux at Eindhoven University of Technology campus. This location offers the opportunity to have an elevated transmitter, on the first-floor balcony of Flux, with a receiver at various locations in and around the parking lot, as shown in Fig. 1. The location consists of a complex and dynamic environment, which includes pedestrians, vehicles, bicycles, vegetation, and buildings during the performance of the experiment. Both LOS and non-line-of-sight (NLOS) conditions can be tested since the adjacent buildings with glass/concrete facades have been found to provide sufficiently strong and coherent reflections^30^.

The transmitter and receiver are placed on carts, as can be seen in Fig. 1d–j. The transmitter cart contains the CO and RAU separated by a spool of fiber, while the receiver cart includes the end-user equipment. The transmitter remains in one geographical location during the entirety of the outdoor measurement campaign; however, the transmitter PAA is manually rotated to achieve different scenarios, as can be seen in Fig. 1a–c. It should be noted that the manual rotation of the transmitter is only needed as an initial step for each scenario, as due to the limit in the beam steering capability of the PAAs not all scenarios are possible for a single broadside position. This emulates a base station setup where multiple PAAs would be needed to support an area of this size or to achieve full 360$^\circ$ coverage. Similarly, the receiver cart is also manually placed and rotated as the initial setup step for the different scenarios prior to the beam steering stage. For the remainder of each scenario all antennas are stationary. Table 2 indicates the manual rotation angles, beam steering angles and the respective distance to the receiver antenna for all scenarios. The beam direction and scanning range of the transmitter are indicated by the red circle sectors, while the receiver’s are indicated by the yellow circle sectors. The dashed red lines show ray-tracing of the main beam’s center at the extremities of the scanning range of the transmitter PAA. The solid lines in Fig. 1a–c indicate the expected LOS and NLOS ray traces.

**Table 2:** Wireless scenario parameters, indicating antenna rotations (with respect to the vertical of Loc. 1) and beam steering angles of transmit (Tx) and receive (Rx) PAA.

Location	Distance (m)	Tx Rot. ($^\circ$)	Tx Beam ($^\circ$)	Rx Rot. ($^\circ$)	Rx Beam ($^\circ$)
1	59.0	0	0.0	0	0.0
2	58.5	0	15.0	15	0.0
3 (LOS)	83.5	−22	22.5	27	–27.5
3 (NLOS)	83.5	−22	−20.0	27	20.0
4	74.2	38	5.0	37	0.0
5	107.3	38	5.0	37	0.0
6	165.5	38	0.0	37	0.0

In Fig. 1a,c, the beam steering capability of the transmitter is tested. From ray-tracing it is determined that these are mainly LOS-only scenarios. It is clear from Fig. 1a that the left most beam direction misses the adjacent building. Though it is possible that a side-lobe from the transmit antenna causes the signal to be received at location 1 (Loc. 1) and possibly also location 2. Location 3 is chosen as a test point for a combined LOS and NLOS scenario, as can be seen from the main traces in Fig. 1b. Location 3 also provides some additional challenges given the location of the tree trunks, shown in Fig. 1g. The ray-tracing shown by the orange line of Fig. 1b indicates LOS is expected for the implemented beam scanning angles of 22.5$^\circ$ and $-27.5^{\circ }$ in transmitter and receiver PAAs, respectively. For the NLOS case, shown by the solid pink line, the transmitter PAA should be set to -20.0$^\circ$ and the receiver PAA to 20.0$^\circ$. For locations 4-6, the environment is expected to yield LOS-only scenarios. Location 5 is located right next to the De Zaale road, on a pedestrian pathway. Location 6 tests the performance at a longer distance of 165.5 m. For all locations and measurements, the transmitter PAA elevation is set to -5$^\circ$, and the receiver PAA is set to 5$^\circ$. Therefore, only azimuth scanning is performed for each scenario.

## Measurement setup and signal processing

This section explains the presented mm-wave IFoF setup at the device and system levels and the associated DSP. First, the configuration and characterization of the pair of PAAs employed to realize beam steering on the experimental testbed is discussed. Second, the utilized mm-wave IFoF wireless scheme is presented and explained, highlighting the used device configurations and the 5G entities that are involved in the test bench. Finally, the key aspects of the employed transmitter and receiver DSP are shown together with the used OFDM configuration.

### Phased array antenna description and characterization

The PAAs used in the setup are antenna panels supplied by NXP Semiconductors which contain 8-by-8 arrays of dual-polarized circular patch antennas. The antenna elements are separated by half a wavelength at 26 GHz, equal to 5.8mm. The panels contain dual polarized beamforming ICs (MMW9014K^31^) which can drive four channels per polarization. Each channel contains a transmit and a receive chain. Each chain has an 8-bit variable gain amplifier and an 8-bit phase shifter, allowing beamsteering in the intended direction and manipulation of the beam shape. The range in gain that can be achieved is $\approx$ 30dB, while the range in phase is from 0$^\circ$ to 360$^\circ$. The array has two ports, one for the horizontal and one for the vertical polarization, which can be operated independently. For optimal performance, calibration of the array is required. The array calibration entails measuring the gain and phase responses of each channel for each gain and phase setting and creating a map of the actual response for any of the 8-bit weights.

The aforementioned array calibration is performed in an anechoic chamber in a near-field setup, where an open-ended waveguide probe is placed at $5\lambda$ distance from the panel. For each channel, the probe is moved directly in front of the associated antenna element, and the gain and phase are swept. For each gain and phase setting, and for each of the 64 elements, the $S_{21}$ (in transmit mode) or $S_{12}$ (in receive mode) parameters are measured. The map is then obtained by extracting the gain and phase of the measured S-parameter at a single frequency point. In this setup, only the channel under test is turned on, while the remaining channels are disabled. This process is sped up by only measuring 8 gain settings and 16 phase settings out of the $256^2$ possible combinations, and interpolating the resulting map.

With the resulting maps, some issues with the PAA can be addressed. Firstly, the gain and phase responses of each channel differ. Furthermore, changing the phase on a channel can lead to an unintended change in gain on that channel and vice-versa. This gain-phase coupling can be addressed by using the interpolated map as a lookup table and selecting the setting that matches the intended response most closely. This method is also used to address the phase offsets between the channels. The gain offsets can only be addressed by scaling the most powerful elements to a lower power level, such that all elements radiate the same amount of power. This power scaling decreases the side-lobe level (SLL) at the cost of gain and radiated power. In order to optimize the link budget, the element powers are not scaled, at the cost of increased SLL.

**Figure 2:**
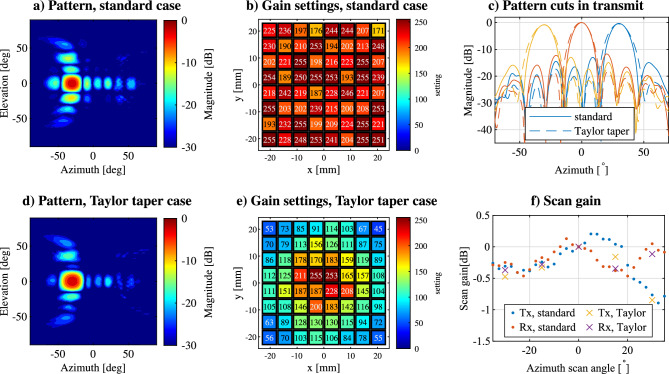
Measured radiation patterns in the standard case when scanning to ${-30}{^\circ }$ in azimuth, normalized to the peak amplitude in (**a**), and the gain settings used to excite the array in (**b**). In (**d**) the pattern is shown when an additional Taylor taper is applied, and the respective gain settings are shown in (**e**). In (**c**) examples of the beam shapes are shown for the transmitter panel using the standard and Taylor taper case. In (**f**) the scanning gain is shown for all measured scanning angles, normalized to the center beam of each situation.

Taking this calibration into account, the phase and gain settings are determined for the transmitter and receiver arrays to steer the beam between ${\pm 35}{^\circ }$ in azimuth in 2.5$^\circ$ steps and ${\pm 5}{^\circ }$ in elevation in ${5}{^\circ }$ steps. This calibration is done in the standard phased array case and for an additional case where a 20dB Taylor taper is applied to the weights, resulting in a reduction in the SLL^32^. These cases are compared in terms of error vector magnitude (EVM) and bit error rate (BER) in the outdoor setup. Compared to the standard configuration, the low-SLL case has a gain reduction of about 4dB both for the transmitter and receiver, resulting in a total 8dB link budget reduction. The radiation patterns generated and their corresponding array settings using the presented method are shown in Fig. 2a,b for the standard case, and in Fig. 2d,e for the low-SLL case. The patterns for Fig. 2a,d are normalized to the maximum gain, in this case in the main beam direction. Here an example is shown for the transmitter case, scanning towards -30$^\circ$ in azimuth and $0^\circ$ in elevation. The patterns are similar in the receiver case. In Fig. 2c the azimuthal cuts of the transmitter radiation patterns are shown in the standard and low-SLL case when scanning from -30$^\circ$ to 30$^\circ$ in azimuth. The different beams in Fig. 2(c) are normalized to the maximum gain at broadside. Observing Fig. 2c, it can be noted that the beamwidth is increased for the low-SLL case by 1.1$^\circ$ and 1.4$^\circ$ for transmit and receive respectively, while the SLL is decreased by 5.1dB and 5.0dB for transmit and receive respectively. In Fig. 2f the gain reduction when scanning, compared to the center beams at 0$^\circ$, is shown, for the transmitter and receiver panels and for standard and low-SLL cases.

**Table 3:** Measured parameters of the phased array antennas.

Array type	Highest scan loss (dB)	Average peak SLL (dB)	HPBW of center beam ($^\circ$)	Integrated gain (dB)
Tx mode standard	0.94	$-10.6$	12.6	47
Rx mode standard	0.55	$-10.9$	11.9	45
Tx mode Taylor	1.07	$-15.7$	13.7	43
Rx mode Taylor	0.72	$-15.9$	13.3	41

The peak SLL averaged across the measured beams for the two under-test configurations, as well as the highest scan loss compared to the center beams at 0$^\circ$ are given in Table 3. The scan loss, as shown in Fig. 2f, is 0.39dB higher in transmit than in receive for the normal configuration, while with Taylor taper this difference reduces to 0.35dB. The Taylor taper reduces the SLL by 5.0dB to 5.1dB and increases the half power beamwidth (HPBW) by 1.1$^\circ$ in transmit and 1.4$^\circ$ in receive direction, respectively. The SLL in the transmitter PAA is almost the same as in the receiver one, and the beamwidths are also equivalent. By using the Friis transmission equation and measuring the cable losses of the anechoic chamber, it is possible to calculate the total integrated gain of the panel for each situation. The integrated gain here is the overall gain of the PAA inclusive of all RF electronics included in the assembly, i.e., array gain, patch gain, splitting losses, beamforming and direction switching, as well as amplification. These results are shown in Table 3 as well. Important to note is that the integrated gain and radiated power are dependent on the array calibration method. The gain difference of the overall PAA assembly in receive and transmit is 2dB. In general, the transmitter and receiver mode arrays perform similarly and their radiation patterns are equivalent.

### Experimental setup

**Figure 3:**
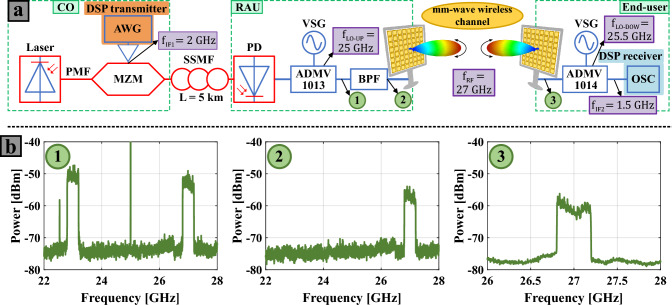
Experimental IFoF wireless setup for mm-wave 5G/6G communications with a single RAU and end-user: (**a**) schematic of the setup (downlink only); (**b**) graphs of the signal spectra at different points of the experimental setup. CO: central office, RAU: remote antenna unit, DSP: digital signal processing, AWG: arbitrary waveform generator, PMF: polarization maintaining fiber, MZM: Mach-Zehnder modulator, SSFM: standard single-mode fiber, PD: photodiode, VSG: vector signal generator, BPF: bandpass filter, OSC: oscilloscope.

Figure 3a depicts the proposed IFoF wireless setup for mm-wave 5G/6G communications. As it can be seen, the schematic of the experimental setup is divided into three different segments which correspond to the different entities of the 5G/6G system^33^: CO, RAU, and end-user. The CO function consists of baseband processing, and generating and preparing the data signal for the optical IFoF fronthaul transport. For achieving this, a distributed-feedback (DFB) laser (CoBrite-DX1 tunable laser from ID Photonics) emits an optical carrier at 1550nm with 16dBm of output power. The optical carrier is then used to convert the electrical data signal into the optical domain by using an Avanex Mach-Zehnder modulator (MZM) (P/N: 792 000220). For proper optical data modulation, the MZM is biased in the quadrature point. The electrical data signal, that is introduced into the MZM, is produced with a 6.4 GSa/s arbitrary waveform generator (AWG). The Zynq UltraScale+ RFSoC ZCU111 evaluation kit is used in this experiment as AWG. The intermediate frequency (IF) upconversion of the baseband data signal is digitally performed in the DSP at 2 GHz and is detailed alongside the DSP. After the optical data modulation, the resulting IF optical data signal is sent through a 5 km long standard single-mode fiber (SSMF), emulating the distance between the CO and the RAU.

At the RAU, the optical signal at the output of the SSMF is detected by a photodiode (PD) from Optilab (P/N: 4323-PD-40-C-DC-ND), generating an electrical bandpass signal at 2 GHz. The resulting electrical signal is upconverted to a center frequency of 27 GHz. For this mm-wave upconversion, a vector signal generator (VSG) and the ADMV1013 evaluation board from Analog Devices are utilized. The ADMV1013 board integrates a local oscillator (LO) quadrupler, RF mixer, and amplification controlled by voltage variable attenuators (VVAs). Hence, the frequency requirements of the VSG are reduced due to the use of the carrier quadrupler. More specifically, the VSG generates a sinusoid of 6.25 GHz. Since the IF mode is the selected configuration on the ADMV1013 board, the upconverted electrical signal is a double-sideband (DSB) with a carrier at 25 GHz (see the spectrum of Fig. 3b1). Moreover, the maximum VVA gain is set in the used ADMV1013 configuration. To condition the output signal for the wireless transmission, a band-pass filter (BPF) is used with a 27 GHz center frequency, $\approx$ 600 MHz of bandwidth and adequate suppression of unwanted and out-of-band components^34^. The spectrum of the signal obtained after this filtering process is illustrated in Fig. 3b2. Next, the filtered signal is fed into the transmitter PAA panel, where splitting, amplification, and phase-shifting processes are carried out for transmit beamforming. Finally, the resulting mm-wave signal is transmitted wirelessly at 27 GHz, within the n257 and n258 5G bands.

After wireless transmission, the PAA panel of the end-user catches the signal, and subsequently, phase shifting, amplification, and combining procedures are performed for receive beamforming. The spectrum of the signal at the output of the receiver PAA can be seen in Fig. 3b3. After the end-user PAA, the mm-wave signal is downconverted to a second IF at 1.5 GHz using the ADMV1014 evaluation board from Analog Devices. A carrier quadrupler, RF mixer, and RF amplifiers are integrated on the ADMV1014 board, which is the complementary downconversion model to the ADMV1013 board used in the RAU. In addition, for this downconversion procedure, a second, independent, VSG is required, which produces an LO at 6.375 GHz. In the presented experiment, two N5183B MXG modules are employed for the transmitter and receiver VSGs. Finally, the resulting IF signal is sampled and captured by an oscilloscope with a sampling rate of 10 GSa/s. The Lecroy WavePro 725Zi is utilized as oscilloscope.

It is relevant to point out that the signal bandwidth of the demonstration system is mainly limited by available spectrum and the filter at the transmitter (600 MHz bandwidth) required to stay within emission limits, while the remainder of the system would support substantially larger bandwidths. The PAAs support signals between 24.0 GHz and 27.5 GHz, i.e., up to 3.5 GHz of bandwidth, while the ADMV1014 and ADMV1013 modules support signals with bandwidths up to 5.2 GHz at IF frequencies between 0.8 GHz and6.0 GHz. Finally, the optical IFoF subsystem would support substantially wider bandwidths and higher IF frequencies.

### DSP configuration

The same OFDM configuration is employed for all the measurements carried out in this work. This OFDM configuration adheres to the 5G standards and is as follows^2^: 14 OFDM symbols per slot; 12 subcarriers per resource block (RB); 240 kHz of subcarrier spacing; every OFDM symbol contains 2048 subcarriers of which 416 are null, resulting in a total bandwidth of 391.68 MHz; one dedicated OFDM symbol per slot for channel estimation with all active subcarriers serving as demodulation reference signals (DM-RSs); one phase tracking reference signal (PT-RS) subcarrier every 8 RB for phase noise compensation^35^; ${0.2976}{\mu }{s}$ of cyclic prefix (CP); and 64-QAM as modulation order on the data subcarriers. With these parameters, the spectral efficiency of the OFDM signal is $0.86{\log }_{2}(M)$ $\hbox {bit/s/Hz}$, where *M* indicates the modulation order. Hence, the final throughput is 2015.5Mbit/s for 64-QAM data modulation and 391.68 MHz of bandwidth.

**Figure 4:**
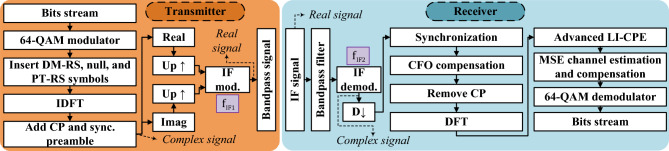
DSP block diagrams for transmitter (left) and receiver (right) sides.

The DSP block diagram used on the transmitter side is represented on the left side of Fig. 4. The resulting signals of this DSP process are generated by the AWG of the RAU (see Fig. 3a). First, in the DSP transmitter block diagram, the input bits are mapped to 64-QAM symbols. The resulting 64-QAM symbols refer to the data subcarriers. Later, null, PT-RS, and DM-RS subcarriers are inserted respecting the OFDM configuration discussed in the previous paragraph. After this subcarrier insertion, an inverse discrete Fourier transform (IDFT) is performed, moving from the frequency to the time domain. Then, the CP is added to each OFDM symbol. All the aforementioned DSP blocks compose the OFDM transmitter. A preamble is also added at the beginning of the 5G slot frame for fine synchronization on the receiver side. Subsequently, the real and imaginary parts of the OFDM signals are separated and upsampled for a 2 GHz IF upconversion in the digital domain. As a result, an OFDM bandpass signal with an IF of 2 GHz is generated.

On the other hand, the block diagram on the right of Fig. 4 corresponds to the DSP processes performed on the receiver side in order to properly demodulate the captured signal by the oscilloscope. The received signal is filtered with a digital BPF, suppressing any undesired frequency components. Then, an IF demodulation procedure is realized, moving the signal to the baseband. The obtained baseband signal is downsampled. By using the preamble previously inserted on the transmitter side, fine synchronization is performed to find the starting time of the received signal. Subsequently, a rough carrier frequency offset (CFO) compensation is executed to correct for the frequency drift of some devices, such as VSGs and AWG, involved in the experimental setup. At this point, the OFDM receiver block starts by removing the CP. For more accurate CFO compensation, the advanced LI-CPE method^36^ is used, harnessing the inserted PT-RS symbols. Furthermore, this LI-CPE method allows efficient mitigation of the common phase error (CPE) induced by the phase noise that equally affects all the subcarriers of each OFDM symbol^36^. After the LI-CPE method, a mean squared error (MSE) channel estimation is carried out by using the DM-RS OFDM symbol contained in every slot^37^. Thus, the MSE detection is utilized to compensate for the channel on the data subcarriers. Finally, a 64-QAM demodulator is employed to extract the bits from the processed data subcarriers.

## Measurement results and discussions

**Figure 5:**
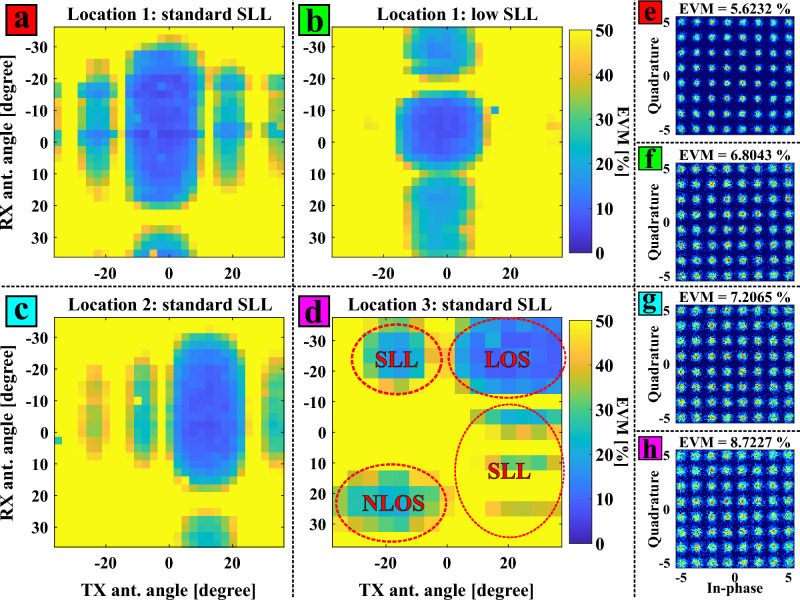
Experimental 2D EVM map results obtained by realizing a double sweep in the transmitter and receiver azimuth beam angles for different end-user locations and system configurations: (**a**) location 1 with standard SLL configuration in the pPAA; (**b**) location 1 with low-SLL configuration; (**c**) location 2 with standard SLL; (**d**) location 3 with standard SLL illustrating LOS and NLOS transmission; (**e**)–(**h**) corresponding constellation diagrams of the minimum EVM points in the results from (**a**)–(**d**).

This section presents and explains the results obtained in the outdoor measurement campaign using the IFoF wireless experimental setup of Fig. 3a. It is important to mention that all the results are collected employing the same DSP, OFDM, and device configurations specified in the previous section. Further, all the measurements in this manuscript were obtained with the same PAA elevation settings: -5$^\circ$ and 5$^\circ$ elevation angles on the transmitter and receiver sides, respectively. In this way, the results of the different measurement locations can be fairly compared and examined. Moreover, the EVM results presented in this manuscript are derived through the calculation of the mean value from the individual EVM values of 20995 64-QAM symbols (see the equation of annex F.2 in^2^).

Figure 5 shows the results of the measurements from locations 1 to 3 of Fig. 1e–g in terms of EVM (Fig. 5a–d) and corresponding constellation diagrams (Fig. 5e–h). These are obtained by processing the end-user oscilloscope traces with the receiver DSP procedure of Fig. 4. The EVM contour maps of Fig. 5a–d are obtained by realizing a double sweep of the beam azimuth angles of the transmitter and receiver PAAs. In other words, the x-axis indicates the transmitter beam azimuth angle while the y-axis corresponds to that of the receiver. For both antennas, the range of the beam angle sweep is $-35 ^{\circ }$ to 35$^\circ$. In addition, the color of these graphs denotes the EVM for the processed 64-QAM data symbols at the input of the QAM demodulator. Cooler colors signify lower EVM; in contrast, warmer colors indicate higher EVM values, with bright yellow color indicating an EVM of 100% or higher.

The results of Fig. 5a,b refer to the same location. However, in these results, the beamforming configuration utilized at the transmitter and receiver sides is different: EVM values of Fig. 5a are obtained with standard SLL configuration, while Fig. 5b is obtained with the low SLL setting. The remaining results in this section are related to the measurements using standard SLL antenna configurations. Moreover, on the right side of Fig. 5, in-phase and quadrature (IQ) constellations of the processed 64-QAM symbols are illustrated. These IQ constellations refer to the minimum EVM points of the graphs in Fig. 5a–d where the EVM value is displayed at the top of the constellation. It is worth mentioning that Fig. 5a–c have a beam angle step of 2.5$^\circ$ while 5$^\circ$ of beam angle step is used for the EVM results of Fig. 5d.

Observing Fig. 5a, it can be noted that the minimum EVM area corresponds to the main lobes of the transmitter and receiver PAA beams. Therefore, the surface of this area should be proportional to the beamwidth of the used PAAs. In other words, the width and height of this area are proportional to the beamwidths of the transmitter and receiver PAAs, respectively. Furthermore, examining Fig. 5a, other low EVM areas are noticed. These EVM spots exhibit higher EVM values and are associated with the SLLs of the employed PAA panels. In order to reduce the interference induced by side lobes for a multi-user scenario case, low SLL configuration is set in both antennas at location 1 of Fig. 1e, obtaining the EVM results of Fig. 5b. Comparing Fig. 5a,b, it can be seen that the blue EVM areas related to the SLLs are less intense in Fig. 5b than in Fig. 5a. This SLL reduction occurs primarily on the transmitter antenna angle axis, while secondary blue EVM spots caused by SLLs still remain in the receiver antenna angle axis. Nonetheless, this interference reduction when using the low SLL configuration leads to a decrease in the maximum received power of $\approx$ 8dB and thus the minimum EVM in Fig. 5a is lower than in Fig. 5b (constellation diagrams for these two measurements are shown in Fig. 5e,f). Therefore, there is a trade-off between SLL reduction to avoid interference with other users and individual user performance.

The results in Fig. 5c correspond to the measurements realized in location 2 (Fig. 1f). For this location the broadside direction of the transmitter PAA remains the same as for location 1, while the broadside direction for the receiver PAA still points at the transmitter although the receiver location has been changed compared to location 1. The receiver for location 2 is shifted to a 15$^\circ$ offset with reference to this transmit broadside direction (see Table 2), hence the shift of the maximum point along the x-axis in Fig. 5c. Otherwise the results for locations 1 and 2 are similar.

On the other hand, Fig. 5d exhibits the EVM results of the measurements carried out in location 3 of Fig. 1b. The main purpose of these measurements is to quantitatively compare LOS and NLOS communications at the same end-user location. As shown in Fig. 1b and Table 2, for this scenario NLOS reception is expected at transmit and receive beam angles of -20$^\circ$ and 20$^\circ$ respectively, since both transmitter and receiver PAAs are rotated manually for initial setup prior to the start of beam steering. Figure 5d shows EVM blue spots with regard to LOS and NLOS links. It is obvious that the EVM values concerning the NLOS communication are higher than in the LOS case: 20.4% of minimum EVM for the NLOS link and 8.7% for the LOS case. These EVM values indicate that the LOS link in this wireless scenario of location 3 permits 64-QAM modulation while the NLOS link conditions are only suitable for QPSK as modulation for the data subcarriers. These results experimentally prove that NLOS communication can be found by properly scanning the wireless scenario with the beamsteering capabilities provided by the employed PAAs. This NLOS link can be used as a secondary channel in case of a blockage of the LOS communication, strengthening the robustness of the mm-wave mobile system by applying algorithms such as beam switching^38^, or to improve performance through joint processing of both LOS and NLOS. In Fig. 5d, there are also low EVM areas caused by the SLLs of the used PAAs. Additionally, the transmitter and receiver antenna angles related to the minimum EVM value of the NLOS are -15$^\circ$ and 25$^\circ$, respectively. Considering the scanning resolution and possible manual alignment errors during outdoor setup, the EVM result match the NLOS link angles considered in the wireless scenario (Fig. 1b, Table 2).

**Figure 6:**
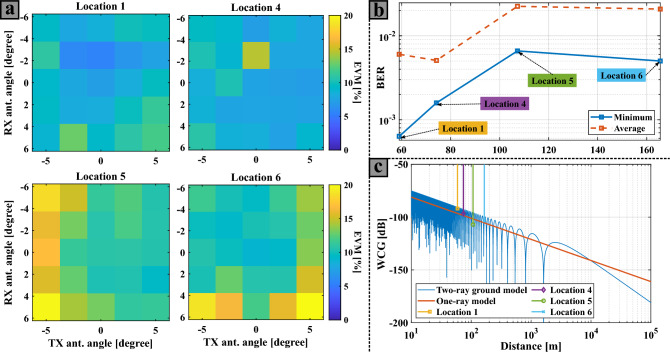
Experimental results in terms of distance: (**a**) 2D EVM map results for different locations where the x-axis and y-axis are the transmitter and receiver antenna beam angles, respectively; (**b**) BER as a function of the distance; (**c**) WCG as a function of the distance by using different ray models where the distances of the under-test locations are also represented.

Figure 6 shows the results obtained from the measurements of locations 1, 4, 5, and 6 (see Fig. 1). In these locations the broadside beams of the transmitter and receiver PAAs point at each other, hence only a small sweep in angles on both PAAs is performed. The aim of Fig. 6 is to represent the performance of the IFoF wireless setup in terms of distance. For that, Fig. 6a depicts 2D EVM color maps referring to the aforementioned locations. In this case, the sweep range of the transmitter and receiver antenna beam angles is -5$^\circ$ to 5$^\circ$, in steps of 2.5$^\circ$. Moreover, in Fig. 6b, the BER results are shown as a function of the distance for each location, with a linear interpolation between the measured data points. In this BER graph, the continuous blue lines represent the minimum value of the complementary BER plots of Fig. 6a, while the dotted orange lines refer to the average of all BER values. Examining Fig. 6b, it is noticeable that location 4 presents the lowest gap between the average and minimum BER results. This gap is related to the impact of the antenna misalignment that can occur in the communication. Thereby, location 4 exhibits more robustness to antenna misalignment than location 1 as the average BER values are lower. The reason for this phenomenon could be that fewer obstacles are involved in the wireless scenario of location 4. The antenna misalignment impact may be estimated based on the maps in Fig. 5 by fixing a specific antenna angle in transmit or receive and calculating the EVM deviation with respect to the minimum EVM value for deviation of the opposite angle. Thus, the offset sensitivity of the antenna angle compared to the ideal aligned angle can be extracted, concluding that locations 5 and 6 suffer from more antenna misalignment impact in terms of EVM and finding EVM impacts of $\approx$  1% and up to 5% for offsets of 2.5$^\circ$ and 5.0$^\circ$ respectively. Also, it is important to notice that the BER results of location 6 are lower than in location 5, even though the link distance is greater in the location 6 case. One of the possible causes of this decrease of BER for larger communication distances is the impact of the ray reflected by the ground is more significant at these distances^39^.

Figure 6c illustrates the theoretical wireless channel gain (WCG) values of the wireless scenarios under-test, considering the one-ray (Friis transmission equation) and two-ray ground-reflection models. Both models assume a carrier frequency of 27 GHz. The two-ray ground-reflected model curve of Fig. 6c is obtained assuming a perfect ground reflection ($\Gamma =1$)^39^. The remaining parameters are as follows: the transmitter and receiver heights ($h_{tx}$ and $h_{rx}$) are 6 m and 1.5 m, respectively. Furthermore, the WCG values of the two-ray ground model for the distances of locations 1, 4, 5, and 6 are also indicated in Fig. 6c. As can be seen, the actual WCG generally decreases with increasing distance, but due to the periodicity of the fading pattern, this is not uniform and higher losses may be observed at shorter distances, which may explain the higher BER at location 5 compared to location 6.

**Table 4:** Summary of the experimental results for the different wireless locations and system configurations. Forward error correction (FEC) thresholds: ${3.8 \times 10^{-3}}$ and ${1.34\times 10^{-2}}$ for 7% and 25% overhead (OH), respectively.

Location	Low SLL	Min (BER)	Min (EVM) (%)	$\varvec{\alpha _{tx}}$ ($^\circ$)	$\varvec{\alpha _{rx}}$ ($^\circ$)	FEC OH (%)	Throughput (Gbit/s)
1	No	$6.0\times {10}^{-4}$	5.6	0	$- 2.5$	7	1.88
1	Yes	$1.5\times {10}^{-3}$	6.8	$- 2.5$	$- 5$	7	1.88
2	No	$1.2\times {10}^{-3}$	7.2	12.5	$- 2.5$	7	1.88
3	No	$2.7\times {10}^{-3}$	8.7	20	$-20$	7	1.88
4	No	$1.6\times {10}^{-3}$	7.2	5	0	7	1.88
5	No	$6.6\times {10}^{-3}$	9.4	5	2.5	25	1.61
6	No	$5.0\times {10}^{-3}$	9.0	0	0	25	1.61

Lastly, Table 4 shows the minimum BER/EVM results for each measurement realized with the presented IFoF wireless experimental setup. The transmitter and receiver antenna beam azimuth angles ($\alpha _{tx}$ and $\alpha _{rx}$) related to these BER/EVM values are also illustrated in Table 4. All these angle values are close to 0$^\circ$, except for the results of locations 2 and 3, because in these scenarios the broadside beams of the PAAs are not aligned (see Fig. 1a,b and Table 2). Moreover, Table 4 indicates the minimum OH percentage for a FEC^40^ that allows a final BER after FEC processing of ${1\times {10}^{-9}}$. Therefore, regarding the throughput value calculated in the previous section, the throughput values taking FEC into account are 1.88 Gbit/s and 1.61 Gbit/s for 7% and 25% of OH FEC, respectively. It is important to mention that a final BER value of ${1\times {10}^{-9}}$ allows a BLER below ${1\times {10}^{-5}}$ for the maximum 5G code block size (8448 bits), fulfilling the most demanding 5G BLER requirement of ${1\times {10}^{-3}}$ of BLER for ultra-reliable and low latency communications (URLLC) scenarios^41^.

## Conclusions

First, in this manuscript, the relevance of ARoF technology is highlighted as a key technology for the future mm-wave 5G/6G fronthaul, with IFoF being a potential candidate because of its attractive benefits. Beamsteering based on PAA is also highlighted as an essential solution for enabling mm-wave mobile communications. An experimental IFoF fronthaul setup for mm-wave wireless transmission is presented and explained in detail. In the proposed experimental setup, 8-by-8 PAA panels with integrated beamsteering and amplification are employed for the transmitter and receiver front-ends, allowing fine beamsteering capabilities in the azimuth and elevation angles. For proper comprehension of the setup, the used PAAs are described and characterized. Furthermore, the configuration of the experimental setup is according to 5G standards, transmitting 64-QAM OFDM signals with $\approx$ 400 MHz of bandwidth at a center frequency of 27 GHz, within the n257 and n258 bands.

A measurement campaign is carried out in different outdoor wireless scenarios, positioning the end-user receiver at different locations and alignment angles with reference to the transmitter. For all these measurements, a double sweep in the azimuth angle of both PAAs is performed in order to evaluate the performance of the experimental setup in different wireless scenarios. Moreover, two different PAA configurations are tested: standard SLL and low SLL. Standard SLL results show lower EVM values while low SLL reduces the user interference induced by SLL. Therefore, there is a clear trade-off between user interference and individual user performance. In addition, LOS and NLOS communications are compared at the same end-user location, permitting a fair comparison between both types of links. The obtained EVM values determine that LOS link is suitable for 64-QAM modulation order while the NLOS link is capable of successfully transmitting data subcarriers with QPSK as modulation format. Thus, in case of a blockage in the LOS communication, it is proven that beamsteering based on PAAs is able to scan a possible NLOS link, increasing the reliability of mm-wave mobile communications.

A comparison of the performance between different end-user locations in terms of link distance is also realized. In this comparison, it is seen that ground reflections might affect the system performance at the distances under test since the obtained BER curve as a function of the distance does not monotonically increase. Overall, excellent EVM and BER performance is observed in all the realized measurement locations, achieving a maximum distance of 165.5 m with a BER below the 25% OH FEC limit and with a final throughput of 1.61 Gbit/s. With the proposed IFoF wireless system and the experimental results, key elements are provided to suitably realize a robust mm-wave wireless fronthaul based on IFoF and PAA technologies. Also, the excellent BER/EVM results obtained in the measurement campaign strengthen wireless IFoF combined with PAA as the preferred solution to transport and transmit high-bandwidth mm-wave signals in future 5G/6G networks.

The achieved results provide an early-stage proof-of-concept system with prototype hardware providing realistic physical layer performance estimates for mm-wave transmission, incl. CCAM use cases for which the PAAs employed would be a realistic form-factor. Further demonstration of full CCAM services and use cases would require further integration of higher layer control functionality as well as related services such as localization and relevant radar and/or lidar sensors.

## Data Availability

The datasets recorded and analyzed during the current study for evaluation of transmission performance are available from the corresponding author on reasonable request. The data regarding the antenna arrays, characterization and beamforming used in this study are available from NXP Semiconductors, but restrictions apply to the availability of these data, which were used with special permission for the current study, and so are not publicly available. Data are available form the authors upon reasonable request and only with explicit permission of NXP Semiconductors.
